# On the combinatorics of sparsification

**DOI:** 10.1186/1748-7188-7-28

**Published:** 2012-10-22

**Authors:** Fenix WD Huang, Christian M Reidys

**Affiliations:** 1 Department of Mathematic and Computer science, University of Southern Denmark, Campusvej 55, DK-5230 Odense M, Denmark

**Keywords:** Sparsification, Generating function, Dynamic programming

## Abstract

**Background:**

We study the sparsification of dynamic programming based on folding algorithms of RNA structures. Sparsification is a method that improves significantly the computation of minimum free energy (mfe) RNA structures.

**Results:**

We provide a quantitative analysis of the sparsification of a particular decomposition rule, Λ^∗^. This rule splits an interval of RNA secondary and pseudoknot structures of fixed topological genus. Key for quantifying sparsifications is the size of the so called candidate sets. Here we assume mfe-structures to be specifically distributed (see Assumption 1) within arbitrary and irreducible RNA secondary and pseudoknot structures of fixed topological genus. We then present a combinatorial framework which allows by means of probabilities of irreducible sub-structures to obtain the expectation of the Λ^∗^-candidate set w.r.t. a uniformly random input sequence. We compute these expectations for arc-based energy models via energy-filtered generating functions (GF) in case of RNA secondary structures as well as RNA pseudoknot structures. Furthermore, for RNA secondary structures we also analyze a simplified loop-based energy model. Our combinatorial analysis is then compared to the expected number of Λ^∗^-candidates obtained from the folding mfe-structures. In case of the mfe-folding of RNA secondary structures with a simplified loop-based energy model our results imply that sparsification provides a significant, constant improvement of 91% (theory) to be compared to an 96% (experimental, simplified arc-based model) reduction. However, we do not observe a linear factor improvement. Finally, in case of the “full” loop-energy model we can report a reduction of 98% (experiment).

**Conclusions:**

Sparsification was initially attributed a linear factor improvement. This conclusion was based on the so called polymer-zeta property, which stems from interpreting polymer chains as self-avoiding walks. Subsequent findings however reveal that the *O*(*n*) improvement is not correct. The combinatorial analysis presented here shows that, assuming a specific distribution (see Assumption 1), of mfe-structures within irreducible and arbitrary structures, the expected number of Λ^∗^-candidates is Θ(*n*^2^). However, the constant reduction is quite significant, being in the range of 96%. We furthermore show an analogous result for the sparsification of the Λ^∗^-decomposition rule for RNA pseudoknotted structures of genus one. Finally we observe that the effect of sparsification is sensitive to the employed energy model.

## Background

### RNA structures, diagrams and genus filtration

An RNA sequence is a linear, oriented sequence of the nucleotides (bases) **A,U,G,C**. These sequences “fold” by establishing bonds between pairs of nucleotides. In this paper, we only consider the Watson-Crick base pair **A-U** or **G-C** and wobble base pairs **U-G**. The global conformation of an RNA molecule is determined by topological constraints encoded at the level of secondary structure, i.e., by the mutual arrangements of the base pairs [1].

**Figure 1:**
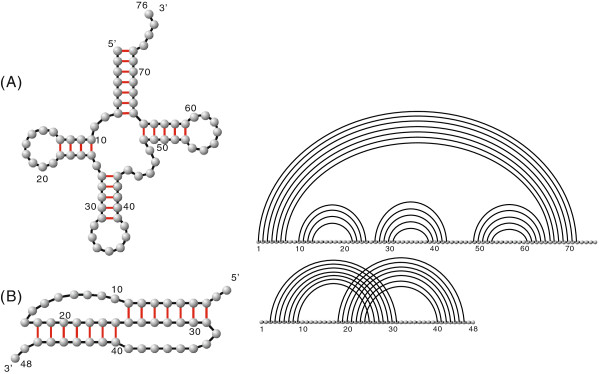
**RNA structures as planar graphs and diagrams.** (**A**) an RNA secondary structure and (**B**) an RNA pseudoknot structure.

Secondary structures can be interpreted as (partial) matchings in a graph of permissible base pairs [2]. They can be represented as diagrams, i.e. graphs over the vertices 1,…,*n*, drawn on a horizontal line with bonds (arcs) in the upper half-plane. The length of an arc (*ij*) is denoted by *j*−*i*. Furthermore, we call two arc (*ij*) and (*rs*) (suppose *i *<* r*) cross if *i*<*r*<*j*<*s* holds. In this representation one refers to a secondary structure without crossing arcs as a *simple* secondary structure and pseudoknot structure, otherwise, see Figure 1.

**Figure 2:**
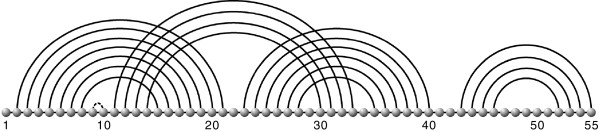
**Diagram representation and irreducibility.** A diagram over {1,…,55}. The arcs (1,21) and (11,33) are crossing and the dashed arc (9,10) is a 1-arc which is not allowed. This structure contains 4 stacks with length 7, 4, 6 and 4, from left to right respectively. Irreducibility relative also to a decomposition rule. The rule Λ^∗^ splitting *S*_*i*,*j*_ to *S*_*i*,*k*_ and *S*_*k* + 1,*j*_, *S*_1,55_ is not Λ^∗^-irreducible, while *S*_2,40_ and *S*_43,55_ are. However, for a specific decomposition rule Λ, which removes the outmost arc, *S*_43,55_ is not Λ-irreducible while *S*_2,40_ is.

A diagram is a labeled graph over the vertex set [*n*]={1,…,*n*} in which each vertex has degree ≤ 3, represented by drawing its vertices in a horizontal line. The backbone of a diagram is the sequence of consecutive integers (1,…,*n*) together with the edges {{*i*,*i* + 1}∣1 ≤ *i* ≤ *n*−1}. The arcs of a diagram, (*i*,*j*), where *i*<*j*, are drawn in the upper half-plane. We shall distinguish the backbone edge {*i*,*i* + 1} from the arc (*i*,*i* + 1), which we refer to as a 1-arc. A stack of length *ℓ* is a maximal sequence of “parallel” arcs, ((*i*,*j*),(*i* + 1,*j*−1),…,(*i* + (*ℓ*−1),*j*−(*ℓ*−1))) and is also referred to as a *ℓ*-stack, see Figure 2.

We shall consider diagrams as fatgraphs, G, that is graphs *G *together with a collection of cyclic orderings, called fattenings. Each fatgraph G determines an oriented surface F(G)[3,4] which is connected if *G* is and has some associated genus *g*(*G*) ≥ 0 and number *r*(*G*)≥1 of boundary components. Clearly, F(G) contains *G* as a deformation retract [5]. Fatgraphs were first applied to RNA secondary structures in [6,7].

A diagram G hence determines a unique surface F(G) (with boundary). Filling the boundary components with discs we can pass from F(G) to a surface without boundary. Euler characteristic, *χ*, and genus, *g*, of this surface is given by *χ *=* v*−*e* + *r* and $g=1-\frac{1}{2}\chi$, respectively, where *v*,*e*,*r* is the number of discs, ribbons and boundary components in G, [5]. The genus of a diagram is that of its associated surface without boundary and a diagram of genus *g* is referred to as *g*-diagram.

A *g*-diagram without arcs of the form (*i*,*i* + 1) (1-arcs) is called a *g*-structure. A *g*-diagram that contains only vertices of degree three, i.e. does not contain any vertices not incident to arcs in the upper half-plane, is called a *g*-matching. A diagram is called irreducible, if and only if it cannot be split into two by cutting the backbone without cutting an arc, see Figure 2.

### Folding algorithms

Folded configurations are energetically somewhat optimal. Here energy is obtained by adding contributions of loops [8] contained in RNA secondary and pseudoknot structures. Any RNA structure has a unique and disjoint decomposition into such loops which are really stems from the fatgraph [9,10] interpretation of such structures in which loops correspond to boundary components [11]. Additional constraints imply further properties, like for instance certain minimum arc-length conditions [12] and the nonexistence of isolated bonds. An mfe-RNA structure can be predicted in polynomials time by means of dynamic programming (DP) routines [12,13].

The most commonly used tools predicting simple RNA secondary structure mfold[13] and the Vienna RNA Package[14], require *O*(*n*^2^) space and *O*(*n*^3^) time. In the following we omit “simple” and refer to secondary structures containing crossing arcs as pseudoknot structures.

Generalizing the matrices of the DP-routines of secondary structure folding [13,14] to gap-matrices [15], leads to a DP-folding of pseudoknotted structures [15] (pknot‐R&E) with *O*(*n*^4^) space an *O*(*n*^6^) time complexity. The following references provide a certainly incomplete list of DP-approaches to RNA pseudoknot structure prediction using various structure classes characterized in terms of recursion equations and/or stochastic grammars: [9,15-26]. The most efficient algorithm for pseudoknot structures is [22] (pknotsRG) having *O*(*n*^2^) space and *O*(*n*^4^) time complexity. This algorithm however considers only a restricted class of pseudoknots.

Note that RNA secondary structures are exactly structures of topological genus zero [27]. The topological classification of RNA structures [10,11,28] has recently been translated into an efficient DP-algorithm [9]. Fixing the topological genus of RNA structures implies that there are only finitely many types, the so called irreducible shadows [11].

### Sparsification

**Figure 3:**
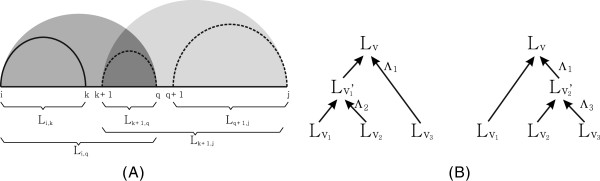
**(A) Sparsification of secondary structure folding.** Suppose the optimal solution *L*_*i*,*j*_ is obtained from the optimal solutions *L*_*i*,*k*_, *L*_*k* + 1,*q*_ and *L*_*q* + 1,*j*_. Based on the recursions of the secondary structures, *L*_*i*,*k*_and *L*_*k* + 1,*q*_ produce an optimal solution of *L*_*i*,*q*_. Similarly, *L*_*k* + 1,*q*_ and *L*_*q* + 1,*j*_ produce an optimal solution of *L*_*k* + 1,*j*_. Now, in order to obtain an optimal solution of *L*_*i*,*j*_ it is sufficient to consider either the grouping *L*_*i*,*q*_ and *L*_*q* + 1,*j*_ or *L*_*i*,*k*_ and *L*_*k* + 1,*j*_. **(B)** General idea of sparsification: *L*_*v*_ is alternatively realized via $L_{v_{1}}$ and $L_{v_{2}^{\prime }}$, or $L_{v_{1}^{\prime }}$ and $L_{v_{3}}$. Thus it is sufficient to only consider one of the computation paths.

Let us have a closer look at sparsification and the results of [29-31]. Sparsification is a method tailored to speed up DP-algorithms predicting mfe-secondary structures [29,31]. The idea is to prune certain computation paths encountered in the DP-recursions, see Figure 3A. Let us consider the case of RNA secondary structure folding. Here sparsification reduces the DP-recursion paths to be based on so called candidates. A candidate is in this case an interval, for which the optimal solution cannot be written as a sum of optimal solutions of sub-intervals. This implies the structure over a candidate is an “irreducible” structures when tracing back from the optimal solution. Considering only these candidates gives the same optimal solution as considering all possible intervals. The crucial observation here is that if these irreducibles appear only at a low rate we have a significant reduction in time and space complexity.

Sparsification has been also applied in the context of RNA-RNA interaction structures [30] as well as RNA pseudoknot structures [32]. In difference to RNA secondary structures, however, not every decomposition rule in the DP-folding of RNA pseudoknot structures is amendable to sparsification. By construction, sparsification can only be applied for calculating mfe-energy structures. Since the computation of the partition function [20,33] needs to take into account *all* sub-structures, sparsification does not work.

Sparsification [29,31,32] can be described as follows: let *V*={*v*_1_*v*_2_,…} be a set whose elements *v*_*i*_are unions of pairwise disjoint intervals. Let furthermore *L*_*v*_ denote an optimal solution (here optimal means to maximize the scores) of the DP-routine over *v*. By assumption *L*_*v*_ is recursively obtained. Suppose we are given a decomposition rule Λ_1_, for which the optimal solution *L*_*v*_ is $L_{v}=L_{v_{1}}+L_{v_{2}}+L_{v_{3}}$, where $v=v_{1}\dot{\cup }v_{2}\dot{\cup }v_{3}$. Then, under certain circumstances, the DP-routine may interpret *L*_*v*_ either as $(L_{v_{1}}+L_{v_{2}})+L_{v_{3}}$ or as $L_{v_{1}}+(L_{v_{2}}+L_{v_{3}})$, see Figure 3B. To be precise, this situation is encountered iff 

there exists an optimal solution $L_{v_{1}^{\prime }}$ for a sub-structure over $v_{1}^{\prime }$ where $v_{1}^{\prime }=v_{1}\dot{\cup }v_{2}$ via Λ_2_ and *L*_*v*_ is obtained from $L_{v_{1}^{\prime }}$ and $L_{v_{3}}$ via Λ_1_,

there exists an optimal solution $L_{v_{2}^{\prime }}$ for a sub-structure over $v_{2}^{\prime }$ where $v_{2}^{\prime }=v_{2}\dot{\cup }v_{3}$ via *Λ*_3_ and *L*_*v*_ is obtained by $L_{v_{1}}$ and $L_{v_{2}^{\prime }}$ via Λ_1_.

Given a decomposition

$$L_{v}=\underset{\Lambda_{1}}{\underset{︸}{\underset{\Lambda_{2}}{\underset{︸}{L_{v_{1}}+L_{v_{2}}}}+L_{v_{3}}}},$$

we call Λ_2_*s*-compatible to Λ_1_ if there exists a decomposition rule Λ_3_ such that

$$L_{v}=\underset{\Lambda_{1}}{\underset{︸}{L_{v_{1}}+\underset{\Lambda_{3}}{\underset{︸}{L_{v_{2}}+L_{v_{3}}}}}}.$$

Note that if Λ_2_ is *s*-compatible to Λ_1_then Λ_3_ is *s*-compatible to Λ_1_. To summarize

#### Definition 1

**(*s*-compatible)** Suppose *L*_*v*_ is the optimal solution for *S*_*v*_ over *v*, $L_{v}=L_{v_{1}^{\prime }}+L_{v_{3}}$ under decomposition rule Λ_1_. $L_{v_{1}^{\prime }}$ is obtained from two optimal solutions $L_{v_{1}}$ and $L_{v_{2}}$ under rule Λ_2_. Then Λ_2_ is called *s-compatible* to Λ_1_ if there exist some rule Λ_3_ such that $L_{v_{2}^{\prime }}=L_{v_{2}}+L_{v_{3}}$ and $L_{v}=L_{v_{1}}+L_{v_{2}^{\prime }}$.

Figure 3B depicts two such ways that realize the same optimal solution *L*_*v*_. Sparsification prunes any such multiple computations of the same optimal value. Note that by symmetry, Λ_2_ and Λ_3_ are both *s*-compatible to Λ_1_.

We next come to the important concept of candidates. The latter mark the essential computation paths for the DP-routine.

#### Definition 2

**(Candidates)** Suppose *L*_*v*_ is an optimal solution in a sense of maximizing. We call *v* is a Λ-*candidate* if for any *v*_1_ ⊊*v* obtained by Λand $\left(v=v_{1}\dot{\cup }v_{2}\right)$, we have

$$L_{v}>L_{v_{1}}+L_{v_{2}}$$

and we shall denote the set of Λ-candidates set by *Q*^Λ^.

By construction a Λ-candidate *v* is a union of disjoint intervals such that its optimal solution *L*_*v*_ cannot be obtained via a Λ-splitting. This optimal solution allows to construct a non-unique arc-configuration (sub-structure) over *v*[13,14] and the above Λ-splitting consequently translates into a splitting of this sub-structure. This connects the notion of Λ-candidates with that of sub-structures and shows that a Λ-candidate implies a sub-structure that is Λ-irreducible.

#### Lemma 1

[29,32] Suppose *L*_*v*_ is obtained by selecting the optimal solution from the decomposition rules Λ_1_Λ_2_,…,Λ_*n*_. If Λ is *s*-compatible to all Λ_*i*_,∀1 ≤ *i * ≤ *n*, then *L*_*v*_ can be obtained via Λ-candidates.

In summary, as for the impact of sparsification, [29] claims that sparsification reduces the time complexity by a linear factor. This claim is based on the assumption that RNA molecules satisfy the *polymer-zeta property*[29]. Subsequent studies draw a slightly different picture [31] concluding that that sparsification requires *O*(*nZ*) time, where *n* denotes the length of input sequence, and *Z* is a sparsity parameter satisfying *n *≤ *Z*<*n*^2^. Recently, it has been shown in [34] that an asymptotic time complexity of a sparsified RNA folding algorithm using standard energy parameters remains *O*(*n*^3^) under a wide variety of condition.

### Sparsification of RNA secondary structures

**Figure 4:**
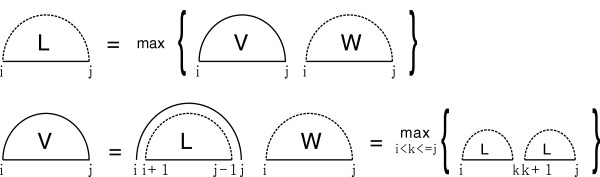
The recursion solving the optimal solution for secondary structures.

Here we recall some results of [29,31] on the sparsification of RNA secondary structures. Secondary structures satisfy a simple recursion which gives the optimal (maximum) solution over *ij* by $L_{i,j}=\max\{V_{i,j},W_{i,j}\}$, where *V*_*i*,*j*_ denotes the optimal solution in which (*ij*) is a base pair, and *W*_*i*,*j*_ denotes the optimal solution obtained by adding the optimal solutions of two subsequent intervals, respectively. Note that the optimal solution over a single vertex is denoted by *L*_*i*,*i*_. We have the recursion equation for *V*_*i*,*j*_ and *W*_*i*,*j*_:

$$\begin{array}{lc} (\Lambda_{1})\;\;V_{i,j}=L_{i+1,j-1}+w(i,j), & \\ (\Lambda_{2})\;W_{i,j}=\underset{i<k<j}{\max}\{L_{i,k}+L_{k+1,j}\}, & \end{array}$$

where *w*(*ij*) is the energy contribution of (*ij*) forming a base pair, see Figure 4. In case two positions, *i*, *j* in the sequence are incompatible then we have *w*(*ij*)=−*∞*.

An interval [*i*,*j*] is a Λ^∗^-candidate if the optimal solution over [*i*,*j*] is given by *L*_*i*,*j*_=*V*_*i*,*j*_>*W*_*i*,*j*_. Indeed, [*i*,*j*] is a candidate iff [*i*,*j*] is in the candidate set of Λ^∗^, and we denote the set $\left(Q^{\Lambda^{*}}\right)$ by *Q*. Suppose the optimal solution *W*_*i*,*j*_ is given by *W*_*i*,*j*_ =* L*_*i*,*q*_ + *L*_*q* + 1,*j*_ and suppose we have *L*_*i*,*q*_ =* L*_*i*,*k*_ + *L*_*k* + 1,*q*_. Then since [*i*,*q*] is not a candidate, Lemma 1 shows that we can compute *W*_*i*,*j*_ =* L*_*i*,*k*_ + *L*_*k* + 1,*j*_, where [*i*,*k*] is a candidate.

### Sparsification on RNA pseudoknot structures

**Figure 5:**
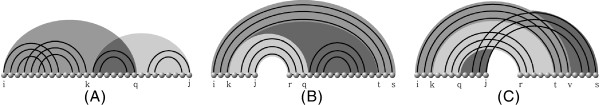
**Decomposition rules for pseudoknot structures of fixed genus (decomposed into three colors).** (**A**) three decompositions via the rule Λ^∗^, which is *s*-compatible to itself. (**B**) three decomposition rules Λ_1_,Λ_2_,Λ_3_where Λ_2_,Λ_3_ are *s*-compatible to Λ_1_. (**C**) three decomposition rules Λ_1_,Λ_2_,Λ_3_ where Λ_2_,Λ_3_ are not *s*-compatible to Λ_1_.

Sparsification can also be applied to the DP-algorithm folding RNA structures with pseudoknots [32]. In contrast to the decomposition rule Λ^∗^ that spliced an interval into two subsequent intervals, we encounter in the grammar for pseudoknotted structures additional more complex decomposition rules [15]. As shown in [32] there exist some decomposition rules which are not *s*-compatible and which can accordingly not be sparsified at all, see Figure 5B. For instance, given a decomposition rule Λ in pknot‐R&E subsequent decomposition rules which are *s*-compatible to Λ are referred to as split type of Λ [32].

In the following we will study RNA pseudoknot structures of fixed topological genus, see **RNA structures, diagrams and genus filtration** for details. An algorithm folding such pseudoknot structures, gfold, has been presented in [9]. The decomposition rules that appear in gfold are reminiscent to those of pknot‐R&E but as they restrict the genus of sub-structures, the iteration of gap-matrices is severely restricted and the effect of sparsification of these decompositions is significantly smaller.

In the following, we restrict our analysis in pseudoknotted structures to only the decomposition rule Λ^∗^, which splices an interval into two subsequent intervals. Put differently, Λ^∗^ cuts the backbone of an RNA pseudoknot structure of fixed genus *g* over one interval without cutting a bond.

### Efficiency of sparsification

By construction, the fewer candidates the DP-routine encounters, the more efficient the sparsification. Thus it is of utmost importance to analyze the number of candidates. In the case of sparsification of RNA secondary structures we have one basic decomposition rule Λ^∗^ acting on intervals, namely Λ^∗^splices an interval into two disjoint, subsequent intervals. The implied notion of a Λ^∗^-irreducible sub-structure is that of a sub-structure nested in a maximal arc, where maximal refers to the partial order of two arcs (*ij*) ≤ (*i*^*′*^*j*^*′*^) iff *i*^*′*^ ≤ *i*∧*j* ≤ *j*^*′*^. This observation relates irreducibility to nesting of arcs and following this line of thought [29] identifies a specific property of polymer-chains introduced in [35,36] to be of relevance for the size of candidate sets:

#### Definition 3

**(Polymer-zeta property)** Let P(i,j) denote the probability of a structure over an interval [*i*,*j*] under some decomposition rule Λ. Then we say Λ follows the polymer-zeta property if $P(i,j)=b\;m^{-c}$ for some constant *b*, *c *> 0 and *m *=* j*−*i*.

Polymer-zeta comes from modeling the 2D-folding of a polymer chain as a self-avoiding walk (SAW) in a 2D lattice [37]. It implies that the probability of a base pair (*ij*) depends only on the length of the arc, i.e. P(i,j)=P(m), where *m *=* j*−*i*. In [29] stipulate that RNA molecules satisfy the polymer-zeta property and approximate P(i,j) by $P(m)=bm^{-c}$[29] using 50,000 mRNA sequences of an average length of 1992 nucleotides [38]. They find *b*≈2.11 and *c*≈1.47. The average probability P(m) is displayed in Figure 4, Page 865 [29] for increasing *m*. Furthermore, it is implied via Figure six, Page 867 [29] that the average number of candidates converges to a constant, implying that sparsification of DP-routine folding secondary structure takes *Λ*(*n*^2^) time complexity.

These findings have been questioned by [34], where it has been observed that the time complexity of a sparsified RNA folding algorithm based on energy minimization remains *O*(*n*^3^) independently of the energy function used and the base composition of the RNA sequence. [34] argues that the significant effect of sparsification on the DP-routine is largely a finite-size effect. Namely, when the sequence length is below some threshold, the algorithm is dominated by the quadratic time factor. In this context, it may be worth pointing out that In [31] noticed that the improvement of a sparsified base-pairing maximization algorithm depends heavily on the base composition of the input. Backofen parameterizes explicitly the cardinality of candidate sets in [31].

### Contribution

In this paper we study the sparsification of the decomposition rule Λ^∗^[31,32] for RNA secondary and RNA pseudoknot structures of fixed topological genus. Based on Assumption 1 below our paper provides a combinatorial framework for quantifying the effects of sparsification of the Λ^∗^ rule.

We shall prove that the candidate set [29,31,32] is indeed small. We compute the probability of an interval being a candidate for two different energy models. For both models, this is facilitated via computing the generating function (GF) of structures and the generating function of irreducible structures. By studying the asymptotics of coefficients in these generating functions, we can compute the expected number of candidates of a uniformly random input sequence for large *n*. We show similar results for RNA pseudoknot structures of fixed topological genus. This provides new insights into the improvements of the sparsification of the concatenation-rule Λ^∗^ in the presence of cross serial interactions. Our observations complement the detailed analysis of Backofen [31,32]. We show that although for pseudoknot structures of fixed topological genus [10,11] the effect of sparsification on the global time complexity is still unclear, the decomposition rule that splits an interval can be sped up significantly.

## Methods

Suppose *w* is an energy function for RNA structures. Let *w*_*δ*_(*σ*) denote the energy of an RNA structure *σ*over a sequence *δ*. The partition function of *δ *is given by

$$Q(\delta )=\underset{\sigma }{\sum }e^{\frac{w_{\delta }(\sigma )}{\text{RT}}},$$

where *R* is the universal gas constant and *T* is the temperature. (Here we consider *w*_*δ*_(*σ*) as a positive score.) The partition function induces a probability space in which the probability of a structure *σ* is

$$P_{\delta }(\sigma )=\frac{e^{\frac{w_{\delta }(\sigma )}{\text{RT}}}}{Q(\delta )}.$$

The concept of a partition function is close to that of a generating function. In case of $\left(e^{w_{\delta }(\sigma )/\text{RT}}=1\right)$, i.e., each structure contributes equally regardless the underlying sequence and the partition function equals [*z*^*n*^]*G*(*z*), where *G* is the generating function and [*z*^*n*^]*G* is the coefficient of the term *z*^*n*^.

Two important energy models are arc-based [39] and loop-based [8], respectively. The loop-based energy-filtration is different from the notion of “stickiness” [40]. The compatibility of two positions by folding random sequences is considered to be 6/16, reminiscent of the probability of two given positions to be compatible by Watson-Crick and Wobble base pairs rules.

### Assumption 1

Let

$$W(\sigma )={\left(\frac{6}{16}\right)}^{\ell }\eta^{w(\sigma )},$$

where *η *> 1 is a constant, *w*(*σ*) is the energy value assigned to *σ* based on a given energy model and *ℓ* is the number of arcs contained in *σ*. Then the probability of a particular structure *σ* to be the mfe-structure of a uniformly random input sequence is

(1) $$P(\sigma )=\frac{W(\sigma )}{\underset{\sigma^{\prime }}{\sum }W(\sigma^{\prime })}.$$

### Asymptotics

In this section we compute two generating functions and their singular expansions [11]. Let **c**_*g*_(*n*) and **d**_*g*_(*n*) denote the number of *g*-matchings and *g*-structures having *n* arcs and *n* vertices, respectively, with GF

$$C_{g}(z)=\overset{\infty }{\underset{n=0}{\sum }}{\text{c}}_{g}(n)z^{n}\;\;{\text{D}}_{g}(z)=\overset{\infty }{\underset{n=0}{\sum }}{\text{d}}_{g}(n)z^{n}.$$

The GF **C**_*g*_(*z*) has been computed in the context of the virtual Euler characteristic of the moduli-space of curves in [41] and **D**_*g*_(*z*) can be derived from **C**_*g*_(*z*) by means of symbolic enumeration [11]. The GF of genus zero diagrams **C**_0_(*z*) is well-known to be the GF of the Catalan numbers, i.e., the numbers of triangulations of a polygon with (*n* + 2) sides,

$$C_{0}(z)=\frac{1-\sqrt{1-4z}}{2z}.$$

As for *g *≥ 1 we have the following situation [11]

#### Theorem 1

Suppose *g *≥ 1. Then the following assertions hold 

(a) **D**_*g*_(*z*) is algebraic and

(2) $$\begin{array}{lcrr} \;D_{g}(z) & = & \frac{1}{z^{2}-z+1}\;C_{g}\left(\frac{z^{2}}{{\left(z^{2}-z+1\right)}^{2}}\right). & \end{array}$$

In particular, $\left(z^{2}/{\left(z^{2}-z+1\right)}^{2}=1/4\right)$ is the only dominant singularity of *D*_*g*_(*z*). we have for some constant *a*_*g*_depending only on *g *and *γ*≈2.618:

(3) $$[z^{n}]D_{g}(z)∼a_{g}\;n^{3(g-\frac{1}{2})}\gamma^{n}.$$

(b) The bivariate GF of *g*-structures over *n* vertices, containing exactly *m* arcs, **E**_*g*_(*z*,*t*), is given by

(4) $$E_{g}(z,t)=\frac{1}{tz^{2}-z+1}D_{g}\left(\frac{t\;z^{2}}{{\left(t\;z^{2}-z+1\right)}^{2}}\right).$$

### Irreducible *g*-structures

In the context of Λ^∗^-candidates we observed that irreducible sub-structures are of key importance. It is accordingly of relevance to understand the combinatorics of these structures. To this end let $D_{g}^{*}(z)=\overset{\infty }{\underset{n=0}{\sum }}{\text{d}}_{g}^{*}(n)z^{n}$ denote the GF of irreducible *g*-structures.

#### Lemma 2

For *g *≥ 0, the GF $\left({\text{D}}_{g}^{*}(z)\right)$ satisfies the recursion

$$\begin{array}{lc} D_{0}^{*}(z)=1-\frac{1}{D_{0}(z)} & \\ D_{g}^{*}(z)=-\frac{\left(D_{0}^{*}(z)-1)D_{g}(z)+\overset{g-1}{\underset{g_{1}=1}{\sum }}D_{g_{1}}^{*}(z)D_{g-g_{1}}(z\right)}{D_{0}(z)}. & \end{array}$$

For a proof of Lemma 2, see Section Proofs.

#### Theorem 2

For *g *≥ 1 we have 

(a) the GF of irreducible *g*-structures over *n*vertices is given by

(5) $$D_{g}^{*}(z)=(z^{2}-z+1)\left(\frac{U_{g}(u)}{{\left(1-4u\right)}^{3g-\frac{1}{2}}}+\frac{V_{g}(u)}{{\left(1-4u\right)}^{3g-1}}\right),$$

where $u=\frac{z^{2}}{{\left(z^{2}-z+1\right)}^{2}}$, **U**_*g*_(*z*) and **V**_*g*_(*z*) are both polynomials with lowest degree at least 2*g*, and **U**_*g*_(1/4), **V**_*g*_(1/4) ≠ 0. In particular, for some constant $\left(a_{g}^{*}>0\right)$ and *γ*≈2.618:

(6) $$D_{g}^{*}(n)∼a_{g}^{*}n^{3\left(g-\frac{1}{2}\right)}\gamma^{n}.$$

(b) the bivariate GF of irreducible *g*-structures over *n* vertices, containing exactly *m* arcs, $\left(E_{g}^{*}(z,t)\right)$, is given by

(7) $$E_{g}^{*}(z,t)\;=\;(tz^{2}-z+1)\left(\frac{U_{g}(v)}{{\left(1-4v\right)}^{3g-\frac{1}{2}}}+\frac{V_{g}(v)}{{\left(1-4v\right)}^{3g-1}}\right)\;,$$

where $v=\frac{tz^{2}}{{\left(tz^{2}-z+1\right)}^{2}}$.

We shall postpone the proof of Theorem 2 to Section Proofs.

### The main result

#### Nussinov-like energy model

In the following we mimic some form of mfe-*g*-structures: inspired by the Nussinov energy model [39] we consider the weight of a *g*-structure over *n* vertices *σ*_*g*,*n*_ to be given by *w*(*σ*_*g*,*n*_) =* cℓ*, where *c* is a constant contribution of a single arc and *ℓ* is the number of arcs in *σ*_*g*,*n*_[40]. Then by Assumption 1, we have the weight function $\left(W(\sigma_{g,n})={\left(6/16\right)}^{\ell }\eta^{c\ell }={\left((6/16)\eta^{c}\right)}^{\ell }\right)$. Note that the case (6/16)*η*^*c*^=1 corresponds to the uniform distribution, i.e. all *g*-structure have identical weight.

This approach requires to keep track of the number of arcs, i.e. we need to employ bivariate GF. In Theorem 1**(b)** we computed this bivariate GF and in Theorem 2**(b)** we derived from this bivariate GF $\left(E_{g}^{*}(z,t)\right)$, the GF of irreducible *g*-structures over *n* vertices containing *ℓ *arcs.

The idea now is to substitute for the second indeterminant, *t*, some fixed $\tau =(6/16)\eta^{c}\in R$. This substitution induces the formal power series

$$D_{g,\tau }(z)=E_{g}(z,\tau ),$$

which we regard as being parameterized by *τ*. Obviously, setting *τ *= 1 we recover **D**_*g*_(*z*), i.e. we have **D**_*g*_(*z*) =** D**_*g*,1_(*z*) =** E**_*g*_(*z*,1). Note that for *τ*>1/4, the polynomial *τz*^2^−*z* + 1 has no real root. Thus we have for *τ*>1/4 the asymptotics

(8) $$\;{\text{d}}_{g,\tau }(n)∼a_{g,\tau }n^{3\left(g-\frac{1}{2}\right)}\gamma_{\tau }^{n}\;\text{and}\;{\text{d}}_{g,\tau }^{*}(n)∼a_{g,\tau }^{*}n^{3\left(g-\frac{1}{2}\right)}\gamma_{\tau }^{n},$$

with identical exponential growth rates as long as the supercritical paradigm [42] applies, i.e. as long as *γ*_*τ*_, the real root of minimal modulus of

$$\left(\frac{\tau \;z^{2}}{{\left(\tau \;z^{2}-z+1\right)}^{2}}\right)=\frac{1}{4},$$

is smaller than any singularity of $\frac{1}{\tau z^{2}-z+1}$. In this situation *τ* affects the constant *a*_*g*,*τ*_ and the exponential growth rate *γ*_*τ*_ but *not* the sub-exponential factor $n^{3(g-\frac{1}{2})}$. The latter stems from the singular expansion of **C**_*g*_(*z*). Analogously, we derive the *τ*-parameterized family of GF $\left(D_{g,\tau }^{*}(z)=E_{g}^{*}(z,\tau )\right)$. We set the contribution of a single arc *c*=1 and the constant *η *=* e*, where *e* is the Euler number. Then we have the parameter *τ *= (6/16)*e*^1^≈1.0125. By abuse of notation we will omit the subscript *τ*assuming *τ *= (6/16)*e*^1^.

The main result of this section is that the set of Λ^∗^-candidates is a small proportion of all entries. To put this size into context we note that the total number of entries considered for the Λ^∗^-decomposition rule is given by

$$M(n)=\overset{n}{\underset{m=1}{\sum }}(n-m+1).$$

##### Theorem 3

Suppose an mfe-*g*-structure over an interval of length *m* is irreducible with probability $\left({\text{d}}_{g}^{*}(m)/{\text{d}}_{g}(m)\right)$, then the expected number of candidates of *g*-structures for sequences of lengths *n* satisfies

$$E_{g}(n)=\Theta (n^{2})$$

and furthermore, setting ${\bar{E}}_{g}(n)=E_{g}(n)/M(n)$ we have

$${\bar{E}}_{g}(n)∼{\text{d}}_{g}^{*}(n)/{\text{d}}_{g}(n)∼b_{g},$$

where *b*_*g*_ > 0 is a constant.

**Figure 6:**
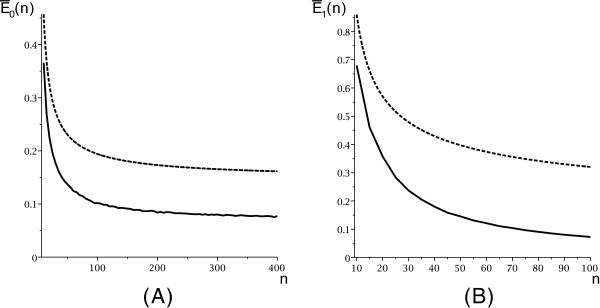
**The expected number of candidates for secondary and 1-structures from an random input with a simplified arc-based energy model,**${\bar{E}}_{0}(n)$**and**${\bar{E}}_{1}(n)$**: we compute the expected number of candidates obtained by folding 100 random sequences for secondary structures (A)(solid) and 1-structures (B)(solid).** We also display the theoretical expectations implied by Theorem 3 (**A**)(dashed) and (**B**)(dashed).

We provide an illustration of Theorem 3 in Figure 6.

##### Proof

We proof the theorem by quantifying the probability of [*i*,*j*] being a Λ^∗^-candidate. In this case any (not necessarily unique) sub-structure, realizing the optimal solution *L*_*i*,*j*_, is Λ^∗^-irreducible, and therefore an irreducible structure over [*i*,*j*].

Let *m *= (*j*−*i* + 1), by assumption, the probability that [*i*,*j*] is a candidate conditional to the existence of a sub-structure over [*i*,*j*] is given by

(9) $$P_{*}\left([i,j]|[i,j]\;\text{is a candidate}\right)\;=\frac{{\text{d}}_{g}^{*}(m)}{{\text{d}}_{g}(m)},$$

Note that $P_{*}\left([i,j]|[i,j]\;\text{is a candidate}\right)$ does not depend on the relative location of the interval but only on the interval-length. Let $P_{g}(m)={\text{d}}_{g}^{*}(m)/{\text{d}}_{g}(m)$, then according to Theorem 1,

$$\begin{array}{lc} (1-\varepsilon )a_{g}m^{3\left(g-\frac{1}{2}\right)}\gamma^{m}\leq {\text{d}}_{g}(m)\;\leq \;(1+\varepsilon )a_{g}m^{3\left(g-\frac{1}{2}\right)}\gamma^{m}, & \\ (1-\varepsilon )a_{g}^{*}m^{3\left(g-\frac{1}{2}\right)}\gamma^{m}\leq {\text{d}}_{g}^{*}(m)\;\leq \;(1+\varepsilon )a_{g}^{*}m^{3\left(g-\frac{1}{2}\right)}\gamma^{m}, & \end{array}$$

for *m *≥* m*_0_ where *m*_0_ > 0 and 0 <* ε *< 1 are constants. On the one hand

(10) $$\begin{array}{l} P_{g}(m)=\frac{{\text{d}}_{g}^{*}(m)}{{\text{d}}_{g}(m)}\leq \frac{(1+\varepsilon )a_{g}^{*}m^{3\left(g-\frac{1}{2}\right)}\gamma^{m}}{(1-\varepsilon )a_{g}m^{3\left(g-\frac{1}{2}\right)}\gamma^{m}}=(1+\varepsilon^{\prime })\frac{a_{g}^{*}}{a_{g}}\\ \;=(1+\varepsilon^{\prime })b_{g}, \end{array}$$

where $\left(b_{g}=a_{g}/a_{g}^{*}>0\right)$ is a constant. On the other hand, we have

(11) $$\begin{array}{l} \;\;\;\;P_{g}(m)=\frac{{\text{d}}_{g}^{*}(m)}{{\text{d}}_{g}(m)}\geq \frac{(1-\varepsilon )a_{g}^{*}m^{3\left(g-\frac{1}{2}\right)}\gamma^{m}}{(1+\varepsilon )a_{g}m^{3\left(g-\frac{1}{2}\right)}\gamma^{m}}=(1-\varepsilon^{\prime \prime })\frac{a_{g}^{*}}{a_{g}}\\ \;=(1-\varepsilon^{\prime \prime })b_{g}. \end{array}$$

**Figure 7:**
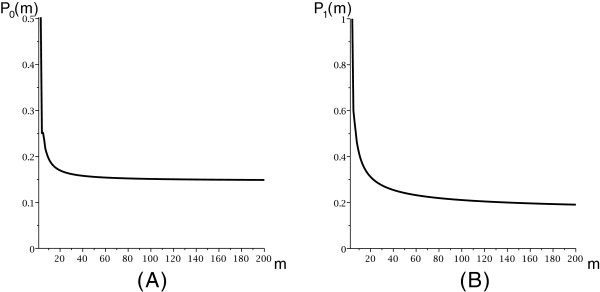
**The probability distribution of**$P_{0}(m)$**(A) and**$P_{1}(m)$**(B) on a simplified arc-based energy model.**

Setting $\varepsilon =\max\{\varepsilon^{\prime },\varepsilon^{\prime \prime }\}$, we can conclude that $P_{g}(m)∼{\text{d}}_{g}^{*}(m)/{\text{d}}_{g}(m)$, see Figure 7.

We next study the expected number of candidates over an interval of length *m*. To this end let

$$X_{m}=|\{[i,j]|[i,j]\;\text{is a}\;\Lambda^{*}\text{-candidate of length}\;m\;\}|.$$

The expected cardinality of the set of Λ^∗^-candidates of length *m *= (*j*−*i* + 1) encountered in the DP-algorithm is given by

$$\begin{array}{ll} E_{g}(X_{m})\leq \;(n-(m-1))\;P_{g}(m), & \end{array}$$

since there are *n*−(*m*−1) starting points for such an interval [*i*,*j*]. Therefore, by linearity of expectation, for sufficiently large *m *>* m*_0_, $P_{g}(m)\leq (1+\varepsilon )b_{g}$ with *ε* being a small constant. Thus we have

(12) $$\begin{array}{l} E_{g}(n)=E_{g}\left(\underset{m}{\sum }X_{m}\right)\;\leq \;\overset{m_{0}}{\underset{m=1}{\sum }}(n\;-\;m\;+\;1)P_{g}(m)\;+\;(1+\varepsilon )b_{g}\\ \;\overset{n}{\underset{m=m_{0}}{\sum }}(n-m+1). \end{array}$$

Consequently, the expected size of the Λ^∗^-candidate set is *Λ*(*n*^2^). We proceed by comparing the expected number of candidates of a sequence with length *n* with M(n),

$$\begin{array}{lll} \;\frac{E_{g}(n)}{M(n)} & \leq \frac{\overset{m_{0}}{\underset{m=1}{\sum }}(n\;-\;m\;+\;1)P_{g}(m)\;+\;(1\;+\;\varepsilon )b_{g}\;\overset{n}{\underset{m=m_{0}}{\sum }}(n\;-\;m\;+\;1)}{\overset{n}{\underset{m=1}{\sum }}(n\;-\;m\;+\;1)}\; & \;\\ & \leq (1+\varepsilon )b_{g}+\frac{\overset{m_{0}}{\underset{m=1}{\sum }}(P_{g}(m)-(1+\varepsilon )b_{g})(n-m+1)}{\overset{n}{\underset{m=1}{\sum }}(n-m+1)}\; & \;\\ & \leq (1+\varepsilon )b_{g}+\frac{k\cdot n}{n^{2}}.\; & \; \end{array}$$

For sufficient large *n *≥ *n*_0_, $E_{g}(n)/M(n)\leq (1+\varepsilon^{\prime })b_{g}$. Furthermore

$$\begin{array}{lll} \;\frac{E_{g}(n)}{M(n)} & \geq \frac{\overset{m_{0}}{\underset{m=1}{\sum }}(n\;-\;m\;+\;1)P_{g}(m)\;+\;(1\;-\;\varepsilon )b_{g}\;\overset{n}{\underset{m=m_{0}}{\sum }}(n\;-\;m\;+\;1)}{\overset{n}{\underset{m=1}{\sum }}(n\;-\;m\;+\;1)}\; & \;\\ & \geq (1-\varepsilon )b_{g},\; & \; \end{array}$$

from which we can conclude $E_{g}\;(n)/M(n)\;∼\;{\text{d}}_{g}^{*}\;(m)/{\text{d}}_{g}(m)∼b_{g}$ and the theorem is proved. □

#### Loop-based energy model

In this section we discuss the loop-based energy model of RNA secondary structure folding. To be precise we evoke here trivariate GFs **F**(*ztv*) and **F**^∗^(*ztv*) whose coefficients counting the numbers of secondary structures and irreducible secondary structures over *n* vertices having *ℓ* arcs and energy *j*, respectively. This becomes necessary since the loop-based model distinguishes between arcs and energy. The “cancelation” effect or reparameterization of stickiness [40] to which we referred to before does not appear in this context. Thus we need both an arc- as well as an energy-filtration.

A further complication emerges. In difference to the GFs **E**_*g*_(*z*,*t*) and $\left(E_{g}^{*}(z,t)\right)$ the new GFs are not simply obtained by formally substituting $\left(\left(tz^{2}/({\left(tz^{2}-z+1\right)}^{2}\right)\right)$ into the power series **D**_*g*_(*z*) and $\left(D_{g}^{*}(z)\right)$ as bivariate terms. The more complicated energy model requires a specific recursion for irreducible secondary structures.

**Figure 8:**
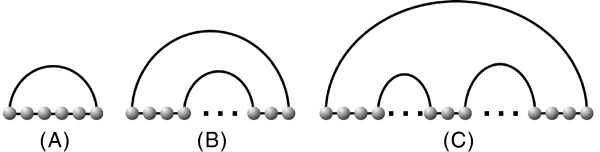
Diagram representation of loop types in secondary structures: (A) hairpin loop, (B) interior loop, (C) multi-loop.

The energy model used in prediction of secondary structure is more complicated than the simple arc-based energy model. Loops which are formed by arcs as well as isolated vertices between the arcs are considered to give energy contribution. Loops are categorized as hairpin loops (no nested arcs), interior loops (including bulge loops and stacks) and multi-loops (more than two arcs nested), see Figure 8. An arbitrary secondary structure can be uniquely decomposed into a collection of mutually disjoint loops. A result of the particular energy parameters [8] is that the energy model prefers interior loops, in particular stacks (no isolated vertex between two parallel arc), and disfavors multi-loops. Based on this observation, we give a simplified energy model for a loop *λ*contained in secondary structure which only depends on the loop types by 

• *w*(*λ*)=0.5 if *λ* is a hairpin loop,

• *w*(*λ*)=1 if *λ* is an interior loop,

• *w*(*λ*)=−5 if *λ* is a multi-loop,

where *λ* is a loop in a structure. The energy for a secondary structure *σ*accordingly is given by

(13) $$w(\sigma )=\underset{\lambda \in \sigma }{\sum }w(\lambda ).$$

Let $\left(F_{0}^{*}(z)\right)$ and **F**_0_(*z*) be the energy-filtered GFs obtained by setting *t*=6/16 and *v*=*η*=*e* in **F**^∗^(*z*,*t*,*v*) and **F**(*z*,*t*,*v*), where *e* is the Euler number. Then

$$\begin{array}{cc} f_{n} & =\underset{\sigma }{\sum }{\left(\frac{6}{16}\right)}^{\ell }e^{w(\sigma )}=\underset{\sigma }{\sum }W(\sigma ),\\ f_{n}^{*} & =\underset{\sigma^{\prime }}{\sum }{\left(\frac{6}{16}\right)}^{\ell^{\prime }}e^{w(\sigma^{\prime })}=\underset{\sigma^{\prime }}{\sum }W(\sigma^{\prime }), \end{array}$$

where *σ* is an arbitary and *σ*^*′*^ is an irreducible secondary structure. Along these lines, *ℓ*, *ℓ*^*′*^ denote the number of arcs in *σ*and *σ*^*′*^. In other words, what happens here is that we find a suitable parameterization which brings us back to a simple univariate GF whose coefficients count the sum of weights of structures over *n* vertices.

##### Lemma 3

The energy-filtered generating function of RNA secondary structures, $\left(F_{0}^{*}(z)\right)$, satisfies the recursion

(14) $$\begin{array}{l} F_{0}^{*}(z)=\frac{6}{16}e^{0.5}z^{2}\frac{z}{1-z}+\frac{6}{16}e^{1}z^{2}{\left(\frac{1}{1-z}\right)}^{2}F_{0}^{*}(z)\\ \;+\frac{6}{16}e^{-5}z^{2}\frac{{\left(F_{0}^{*}(z)\frac{1}{1-z}\right)}^{2}}{1-{\text{F}}_{0}^{*}(z)\frac{1}{1-z}}\frac{1}{1-z}. \end{array}$$

and **F**^∗^(*z*) is uniquely determined by the above equation. Furthermore

(15) $$F_{0}(z)=\frac{1}{1-z}\frac{1}{1-F_{0}^{*}(z)\frac{1}{1-z}}.$$

##### Proof

We first consider the GF $\left(F_{0}^{*}(z)\right)$ whose coefficient of *z*^*n*^ denotes the total weight of irreducible secondary structures over *n* vertices, where (1,*n*) is an arc. Thus it gives a term 6/16*z*^2^. Isolated vertex lead to the term

$$z^{p}\overset{\infty }{\underset{i=0}{\sum }}z^{i}=z^{p}\frac{1}{1-z},$$

where *p* denotes the minimum number of isolated vertices to be inserted. Depending on the types of loops formed by (*i*,*n*), we have 

• hairpin loops: $\frac{z}{1-z}$,

• interior loops: $F_{0}^{*}(z){\left(\frac{1}{1-z}\right)}^{2}$,

• multi-loops: there are at least two irreducible sub-structures, as well as isolated vertices, thus

$$\frac{1}{1-z}\overset{\infty }{\underset{i=2}{\sum }}{\left(F_{0}^{*}(z)\frac{1}{1-z}\right)}^{i}=\frac{{\left(F_{0}^{*}(z)\frac{1}{1-z}\right)}^{2}}{1-F_{0}^{*}(z)\frac{1}{1-z}}\frac{1}{1-z}.$$

Considering the contributions from the energy model we compute

$$\begin{array}{c} F_{0}^{*}(z)=\frac{6}{16}\left(\;e^{0.5}z^{2}\frac{z}{1-z}+e^{1}z^{2}{\left(\frac{1}{1-z}\right)}^{2}F_{0}^{*}(z)\right)\\ \;+\left(e^{-5}z^{2}\frac{{\left(F_{0}^{*}(z)\frac{1}{1-z}\right)}^{2}}{1-F_{0}^{*}(z)\frac{1}{1-z}}\frac{1}{1-z}\right), \end{array}$$

which establishes the recursion. The uniqueness of the solution as a power series follows from the fact that each coefficient can evidently be recursively computed.

An arbitrary secondary structure can be considered as a sequence of irreducible sub-structures with certain intervals of isolated vertices. Thus

$$F_{0}(z)=\frac{1}{1-z}\overset{\infty }{\underset{i=0}{\sum }}\frac{1}{1-z}F_{0}^{*}(z)=\frac{1}{1-z}\frac{1}{1-F_{0}^{*}(z)\frac{1}{1-z}}.$$

□

##### Lemma 4

$\left(F_{0}^{*}(z)\right)$ and **F**_0_(*z*) have the same singular expansion.

(16) $$f_{0}^{*}(n)∼\alpha n^{-\frac{3}{2}}\gamma^{n},\;\text{and}\;f_{0}(n)∼\beta n^{-\frac{3}{2}}\gamma^{n},$$

where *α*≈0.24 and *β*≈2.88 are constants and *γ*≈2.1673

##### Proof

Solving eq. 14 we obtain a unique solution for $\left(F_{0}^{*}(z)\right)$ whose coefficient are all positive. Observing the dominant singularity of $\left(F_{0}^{*}(z)\right)$ is *ρ*≈0.4614. **F**_0_(*z*) is a function of $\left(F_{0}^{*}(z)\right)$ and we examine the real root of minimal modulus of $1-F_{0}^{*}(z)\frac{1}{1-z}=0$ is bigger than *ρ*. Then by the supercritical paradigm [42] applying, **F**_0_(*z*) and $\left(F_{0}^{*}(z)\right)$ have identical exponential growth rates. Furthermore, $\left(F_{0}^{*}(z)\right)$ and **F**_0_(*z*) have the same sub-exponential factor $n^{-\frac{3}{2}}$, hence the lemma. □

##### Theorem 4

Suppose an mfe-secondary structure over an interval of length *m* is irreducible with probability $P_{0}(m)=\frac{f_{0}^{*}(m)}{f_{0}(m)}$, then the expected number of candidates from a random sequence of length *n* with a simplified loop-based energy model is

$$E_{0}(n)=\Theta (n^{2})$$

and furthermore, setting ${\bar{E}}_{g}(n)=E_{g}(n)/M(n)$, we have

$${\bar{E}}_{0}(n)∼f_{0}^{*}(n)/f_{0}(n)∼b,$$

where *b*=*α*/*β*≈0.08.

##### Proof

By Lemma 4 we have $\left(f_{0}^{*}(m)/f_{0}(m)∼b\right)$ where *b* is a constant. The proof is completely analogous to that of Theorem 3. □

**Figure 9:**
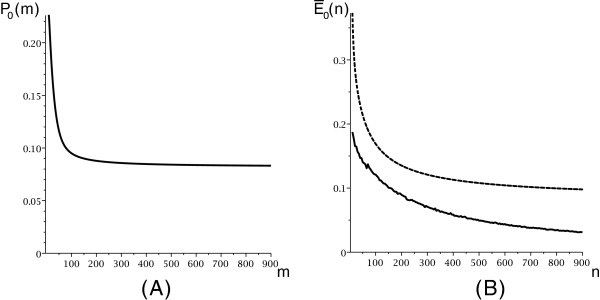
**The distribution of**$P_{0}(m)$**(A) and**${\bar{E}}_{0}(n)$**obtained by folding 100 random sequences on the loop-based model (B)(solid), as well as the theoretical expectation implied by Theorem** 4 **(B)(dashed).**

We show the distribution of $P_{0}(m)$ and ${\bar{E}}_{0}(n)$ in Figure 9.

## Conclusion

In this paper we quantify the effect of sparsification of the rule Λ^∗^. This rule splits intervals and separates concatenated sub-structures. The sparsification of Λ^∗^ alone is claimed to provide a speed up of up to a linear factor of the DP-folding of RNA secondary structures [29]. A similar conclusion is drawn in [30] where the sparsification of RNA-RNA interaction structures is shown to experience also a linear reduction in time complexity. Both papers [29,30] base their conclusion on the validity of the polymer-zeta property. However, [34] comes to a different conclusion reporting a mere constant reduction in time complexity. While Λ^∗^is the key for the time complexity reduction of secondary structure folding, it is conceivable that for pseudoknot structures there may exist non-sparsifiable rules in which case the overall time complexity is not reduced.

In any case, the key is the set of candidates and we provide an analysis of Λ^∗^-candidates by combinatorial means. In general, the connection between candidates, i.e. unions of disjoint intervals and the combinatorics of structures is actually established by the algorithm itself via backtracking: at the end of the DP-algorithm a structure is being generated that realizes the previously computed energy as mfe-structure. This connects intervals and sub-structures.

So, does the condition *c*>1 in polymer-zeta apply in the context of RNA structures? In fact this condition would follow *if * the intervals in question are distributed as in uniformly sampled structures. This however, is far from reasonable, due to the fact that the mfe-algorithm deliberately designs some mfe-structure over the given interval. What the algorithm produces is in fact antagonistic to uniform sampling. We here wish to acknowledge the help of one anonymous referee in clarifying this point.

Our results imply that polymer-zeta does not hold. Our framework critically depends on a specific distribution of mfe structures within irreducible and arbitrary structures, explicated in Assumption 1. We have cross-checked Assumption 1 with the number of candidates in DP-programs (using the same energy model), see Figure 7 and Figure 9. With this conclusion we are in accord with [31,34] but provide an entirely different approach.

The non validity of polymer-zeta has also been observed in the context of the limit distribution of the 5’-3’ distances of RNA secondary structures [43]. Here it is observed that long arcs, to be precise arcs of lengths *O*(*n*)* always* exist. This is of course a contradiction to the polymer-zeta property in case of *c*>1.

The key to quantification of the expected number of candidates is the singularity analysis of a pair of energy-filtered GF, namely that of a class of structures and that of the subclass of all such structures that are irreducible. We show that for various energy models the singular expansions of both these functions are essentially *equal*–modulo some constant. This implies that the expected number of candidates is *Λ*(*n*^2^) and all constants can explicitly be computed from a detailed singularity analysis. The good news is that depending on the energy model, a significant constant reduction, around 96% can be obtained. This is in accordance with data produced in [31] for the mfe-folding of random sequences. There a reduction by 98% is reported for sequences of length ≥ 500.

Our findings are of relevance for numerous results, that are formulated in terms of sizes of candidate sets [32]. These can now be quantified. It is certainly of interest to devise a full fledged analysis of the loop-based energy model. While these computations are far from easy our framework shows how to perform such an analysis.

Using the paradigm of gap-matrices Backofen has shown [32] that the sparsification of the DP-folding of RNA pseudoknot structures exhibits additional instances, where sparsification can be applied, see Figure 5B. Our results show that the expected number of candidates is *Λ*(*n*^2^), where the constant reduction is around 90%. This is in fact very good new since the sequence length in the context of RNA pseudoknot structure folding is in the order of hundreds of nucleotides. So sparsification of further instances does have an significant impact on the time complexity of the folding.

## Proofs

In this section, we prove Lemma 2 and Theorem 2.

**Proof for Lemma 2:** let **D**(*z*,*u*) and **D**^∗^(*z*,*u*) be the bivariate GF $D(z,u)=\underset{n\geq 0}{\sum }\overset{\left\lfloor \frac{n}{2}\right\rfloor }{\underset{g=0}{\sum }}{\text{d}}_{g}(n)z^{n}u^{g}$, and $D^{*}(z,u)=\underset{n\geq 1}{\sum }\overset{\left\lfloor \frac{n}{2}\right\rfloor }{\underset{g=0}{\sum }}{\text{d}}_{g}^{*}(n)z^{n}u^{g}$. Suppose a structure contains exactly *j* irreducible structures, then

(17) $$\text{D}(z,u)=\underset{j\geq 0}{\sum }R{\left(z,u\right)}^{j}=\frac{1}{1-R(z,u)}$$

and

(18) $$D_{g}^{*}(z)=[u^{g}]D^{*}(z,u)=-[u^{g}]\frac{1}{D(z,u)},\;g\geq 1,$$

as well as $D_{0}^{*}(z)=1-\;[u^{0}]\frac{1}{D(z,u)}$. Let $F(z,u)=\underset{n\geq 0}{\sum }$$\underset{g\geq 0}{\sum }\;f_{g}\;(n)\;z^{n}\;u^{g}=\frac{1}{D(z,u)}$. Then *F*(*z*,*u*)*D*(*z*,*u*)=1, whence for *g *≥ 1,

(19) $$\overset{g}{\underset{g_{1}=0}{\sum }}F_{g_{1}}(z)D_{g-g_{1}}(z)=[u^{g}]F(z,u)D(z,u)=0,$$

and *F*_0_(*z*)*D*_0_(*z*)=1, where $F_{g}(z)=\underset{n\geq 0}{\sum }f_{g}(n)z^{n}=[u^{g}]F(z,u)=\;[u^{g}]\frac{1}{D(z,u)}$. Furthermore, we have $F_{0}(z)=\frac{1}{D_{0}(z)}$ and

(20) $${\text{F}}_{g}(z)=-\frac{\overset{g-1}{\underset{g_{1}=0}{\sum }}F_{g_{1}}(z)D_{g-g_{1}}(z)}{D_{0}(z)},\;g\geq 1,$$

which implies $D_{0}^{*}(z)=1-F_{0}(z)=1-\frac{1}{D_{0}(z)}$ and

(21) $$\begin{array}{l} D_{g}^{*}(z)=-F_{g}(z)\\ \;=-\frac{\left(D_{0}^{*}(z)-1)D_{g}(z)+\overset{g-1}{\underset{g_{1}=1}{\sum }}D_{g_{1}}^{*}(z)D_{g-g_{1}}(z\right)}{D_{0}(z)}. \end{array}$$

**Proof for Theorem 2** Let [*n*]_*k*_denote the set of compositions of *n* having *k* parts, i.e. for *σ*∈[*n*]_*k*_ we have *σ*=(*σ*_1_,…,*σ*_*k*_) and $\overset{k}{\underset{i=1}{\sum }}\sigma_{i}=n$.*Claim.*

(22) $$\begin{array}{c} D_{g+1}^{*}(z)=\frac{D_{g+1}(z)}{D_{0}{\left(z\right)}^{2}}+\overset{g-1}{\underset{j=0}{\sum }}\frac{{\left(-1\right)}^{g+2-j}}{D_{0}{\left(z\right)}^{g+2-j}}\\ \;\times \left(\underset{\sigma \in {\left[g+1\right]}_{g+1-j}}{\sum }\overset{g+1-j}{\underset{i=1}{\prod }}D_{\sigma_{i}}(z)\right). \end{array}$$

We shall prove the claim by induction on *g*. For *g*=1 we have

(23) $$D_{1}^{*}(x)=\frac{D_{1}(z)}{{\left(D_{0}(z)\right)}^{2}},$$

whence eq. (22) holds for *g*=1. By induction hypothesis, we may now assume that for *j* ≤ *g*, eq. (22) holds. According to Lemma 2, we have

$$\begin{array}{lll} \;D_{g+1}^{*}(z) & =-\frac{\left(D_{0}^{*}(z)\;-\;1)D_{g+1}\;(z)\;+\;\;\overset{g}{\underset{g_{1}=1}{\sum }}D_{g_{1}}^{*}\;(z)D_{g+1-g_{1}}\;(z\right)}{D_{0}(z)}\; & \;\\ & =\frac{D_{g+1}(z)}{D_{0}{\left(z\right)}^{2}}-\overset{g}{\underset{g_{1}=1}{\sum }}\left(\frac{D_{g_{1}}(z)}{D_{0}{\left(z\right)}^{3}}+\overset{g_{1}-2}{\underset{j=0}{\sum }}\frac{{\left(-1\right)}^{g_{1}+1-j}}{D_{0}{\left(z\right)}^{g_{1}+2-j}}\right)\; & \;\\ & \;\times \left(\left(\underset{\sigma \in {\left[g_{1}\right]}_{g_{1}-j}}{\sum }\overset{g_{1}-j}{\underset{i=1}{\prod }}D_{\sigma_{i}}(z)\right)\right)D_{g+1-g_{1}}(z).\; & \; \end{array}$$

We next observe

(24) $$\begin{array}{l} -\overset{g}{\underset{g_{1}=1}{\sum }}\frac{D_{g_{1}}\;(z)}{D_{0}{\left(z\right)}^{3}}D_{g+1-g_{1}}(z)\\ =\frac{{\left(-1\right)}^{g+2-(g-1)}}{D_{0}{\left(z\right)}^{g+2-(g-1)}}\left(\underset{\sigma^{\prime }\in {\left[g+1\right]}_{g+1-(g-1)}}{\sum }\overset{g+1-(g-1)}{\underset{i=1}{\prod }}D_{\sigma_{i}^{\prime }}(z)\right), \end{array}$$

and setting *h*=*g*_1_−*j* we obtain,

$$\begin{array}{ll} -\overset{g}{\underset{g_{1}=1}{\sum }}\overset{g_{1}-2}{\underset{j=0}{\sum }}\frac{{\left(-1\right)}^{g_{1}+1-j}}{D_{0}{\left(z\right)}^{g_{1}+2-j}}\;\left(\underset{\sigma \in {\left[g_{1}\right]}_{g_{1}-j}}{\sum }\overset{g_{1}-j}{\underset{i=1}{\prod }}D_{\sigma_{i}}(z)\right)\;D_{g+1-g_{1}}(z) & \\ =\overset{g}{\underset{g_{1}=1}{\sum }}\overset{g_{1}}{\underset{h=2}{\sum }}\frac{{\left(-1\right)}^{h+2}}{D_{0}{\left(z\right)}^{h+2}}\left(\underset{\sigma \in {\left[g_{1}\right]}_{h}}{\sum }\overset{h}{\underset{i=1}{\prod }}D_{\sigma_{i}}(z)\right)D_{g+1-g_{1}}(z) & \\ =\overset{g}{\underset{h=2}{\sum }}\frac{{\left(-1\right)}^{h+2}}{D_{0}{\left(z\right)}^{h+2}}\left(\overset{g}{\underset{g_{1}=h}{\sum }}\left(\underset{\sigma \in {\left[g_{1}\right]}_{h}}{\sum }\overset{h}{\underset{i=1}{\prod }}D_{\sigma_{i}}(z)\right)D_{g+1-g_{1}}(z)\right) & \\ =\overset{g}{\underset{h=2}{\sum }}\frac{{\left(-1\right)}^{h+2}}{D_{0}{\left(z\right)}^{h+2}}\left(\underset{\sigma^{\prime }\in {\left[g+1\right]}_{h+1}}{\sum }\overset{h+1}{\underset{i=1}{\prod }}D_{\sigma_{i}^{\prime }}(z)\right) & \end{array}$$

and setting *j*=*g*−*h*

$$=\overset{g-2}{\underset{j=0}{\sum }}\frac{{\left(-1\right)}^{g+2-j}}{D_{0}{\left(z\right)}^{g+2-j}}\left(\underset{\sigma^{\prime }\in {\left[g+1\right]}_{g+1-j}}{\sum }\overset{g+1-j}{\underset{i=1}{\prod }}D_{\sigma_{i}^{\prime }}(z)\right).$$

Consequently, the Claim holds for any *g *≥ 1.

For any *g *≥ 1, we have [11]

$$\begin{array}{l} D_{g}(z)=\frac{1}{z^{2}-z+1}\frac{{\text{P}}_{g}(u)}{{\left(1-4u\right)}^{3g-1/2}},\\ D_{0}(z)=\frac{1}{z^{2}-z+1}\frac{2}{\left(1+\sqrt{1-4u}\right)}, \end{array}$$

where **P**_*g*_(*u*) is a polynomial with integral coefficients of degree at most (3*g*−1), **P**_*g*_(1/4)≠0, *u*^2*g*^**P**_*g*_(*u*)≠0 and *u*^*h*^**P**_*g*_(*u*)=0 for 0 ≤ *h* ≤ 2*g*−1. Let $u=\frac{z^{2}}{{\left(z^{2}-z+1\right)}^{2}}$, the Claim provides in this context the following interpretation of $\left(D_{g}^{*}(z)\right)$

(56) $$\begin{array}{l} \frac{1}{z^{2}-z+1}D_{g}^{*}(z)=\frac{{\text{P}}_{g}(u)}{{\left(1-4u\right)}^{3g-1/2}}{\left(\frac{1+\sqrt{1-4u}}{2}\right)}^{2}\\ \;+\overset{g-2}{\underset{j=0}{\sum }}{\left(-\frac{1+\sqrt{1-4u}}{2}\right)}^{g+1-j}\\ \;\times \frac{\underset{\sigma \in {\left[g\right]}_{g-j}}{\sum }\overset{g-j}{\underset{i=1}{\prod }}{\text{P}}_{\sigma_{i}}(u)}{{\left(1-4u\right)}^{3g-\frac{g-j}{2}}}, \end{array}$$

and

∑j=0g−2−1+1−4u2g+1−j∑σ∈[g]g−j∏i=1g−jPσi(u)(1−4u)3g−g−j2=∑j=0g−2∑k=0g+1−j−12g+1−jg+1−jk∑σ∈[g]g−j∏i=1g−jPσi(u)(1−4u)3g−g−j+k2=∑j=0g−2∑s=g−j2g+1−2j−12g+1−jg+1−js−g+j∑σ∈[g]g−j∏i=1g−jPσi(u)(1−4u)3g−s2.

As 0 ≤ *j* ≤ *g*−2 and *g*−*j* ≤ *s* ≤ 2*g* + 1−2*j*, we have *s *≥ 2. Consequently we arrive at

(26) $$\frac{1}{z^{2}-z+1}D_{g}^{*}(z)=\frac{U_{g}(u)}{{\left(1-4u\right)}^{3g-1/2}}+\frac{V_{g}(u)}{{\left(1-4u\right)}^{3g-1}},$$

where

Ug(u)=Pg(u)4+Pg(u)(1−4u)4+∑j=0g−2∑g−j≤s≤2g+1−2jsis odd×−12g+1−jg+1−js−g+j∑σ∈[g]g−j∏i=1g−jPσi(u)×(1−4u)s−12,

and

Vg(u)=Pg(u)2+−123∑σ∈[g]2∏i=12Pσi(u)+3−123×∑σ∈[g]2∏i=12Pσi(u)(1−4u)+∑j=0g−3∑g−j≤s≤2g+1−2jsis even×−12g+1−jg+1−js−g+j∑σ∈[g]g−j∏i=1g−jPσi(u)×(1−4u)s−22.

We have for *σ*∈[*g*]_*k*_, *k* ≥ 1

$$[u^{h}]\left(\underset{\sigma \in {\left[g\right]}_{k}}{\sum }\overset{k}{\underset{i=1}{\prod }}{\text{P}}_{\sigma_{i}}(u)\right)=\underset{\sigma \in {\left[g\right]}_{k}}{\sum }\overset{k}{\underset{i=1}{\prod }}[u^{h_{i}}]{\text{P}}_{\sigma_{i}}(u),$$

where $\overset{k}{\underset{i=1}{\sum }}h_{i}=h$, *h*_*i*_ ≥ 0. Then we obtain that

(27) $$[u^{h}]\left(\underset{\sigma \in {\left[g\right]}_{k}}{\sum }\overset{k}{\underset{i=1}{\prod }}{\text{P}}_{\sigma_{i}}(u)\right)=0,\;0\leq h\leq 2g-1.$$

Since $[u^{h_{i}}]{\text{P}}_{\sigma_{i}}(u)=0$, *h*_*i*_≤2*σ*_*i*_−1, $[u^{2\sigma_{i}}]{\text{P}}_{\sigma_{i}}(u)\neq 0$ and $\overset{k}{\underset{i=1}{\sum }}\sigma_{i}=g$. Thus for 0 ≤ *h* ≤ 2*g*−1,

(28) $$[u^{h}]U_{g}(u)=0\;\text{and}\;[u^{h}]V_{g}(u)=0.$$

As shown in [11] we have

(29) $${\text{P}}_{g}(1/4)=\frac{\Gamma \left(g-1/6\right)\Gamma \left(g+1/2\right)\Gamma \left(g+1/6\right)9^{g}4^{-g}}{6\Pi^{3/2}\Gamma \left(g+1\right)}$$

and we obtain **U**_*g*_(1/4)=**P**_*g*_(1/4)/4. Furthermore,

$$\begin{array}{l} V_{g}(1/4)=\frac{{\text{P}}_{g}(1/4)}{2}+{\left(-\frac{1}{2}\right)}^{3}\left(\underset{\sigma \in {\left[g\right]}_{2}}{\sum }\overset{2}{\underset{i=1}{\prod }}{\text{P}}_{\sigma_{i}}(1/4)\right)\\ \;=\frac{1}{8}\left(4{\text{P}}_{g}(1/4)-\overset{g-1}{\underset{j=1}{\sum }}{\text{P}}_{j}(1/4){\text{P}}_{g-j}(1/4)\right)\neq 0. \end{array}$$

We can recruit the computation of [11] in order to observe $4{\text{P}}_{g}(1/4)-\overset{g-1}{\underset{j=1}{\sum }}{\text{P}}_{j}(1/4){\text{P}}_{g-j}(1/4)\neq 0$. In order to compute the bivariate GF, $\left(E_{g}^{*}(z,t)\right)$, we only need to replace in eq. (22) **D**_*g*_(*z*) by **E**_*g*_(*zt*) and the proof is completely analogous.

## Competing interests

The authors declare that they have no competing interests.

## Authors’ contributions

FWDH and CMR contributed equally to research and manuscript. Both authors read and approved the final manuscript.
